# SARS-CoV-2: An Analysis of the Vaccine Candidates Tested in Combatting and Eliminating the COVID-19 Virus

**DOI:** 10.3390/vaccines10122086

**Published:** 2022-12-07

**Authors:** Laila Elmancy, Hala Alkhatib, Anis Daou

**Affiliations:** Pharmaceutical Sciences Department, College of Pharmacy, QU Health, Qatar University, Doha P.O. Box 2713, Qatar

**Keywords:** severe acute respiratory syndrome coronavirus 2, SAR-CoV-2, pandemic, vaccines, vaccine candidates, clinical trials, WHO

## Abstract

Severe Acute Respiratory Syndrome coronavirus 2 (SARS-CoV-2), better known as COVID-19, is a highly contagious virus, transferable via air droplets from close human-human contact. The pandemic has led to over 6.5 million deaths worldwide, making it the largest global health crisis since the influenza pandemic in 1918. SARS-CoV-2 rapidly spread around the world, forcing the World Health Organization (WHO) to deem it a global health pandemic after three months of its initiation. The virus has wreaked havoc on many countries worldwide, overwhelming healthcare systems, hence damaging many economies. Even though research has progressed the understanding of the SARS-CoV-2 virus, the information gathered about the vaccine trials and their findings have been scarcely distributed to the public in a single study. The information available to scientists has therefore given researchers a pathway to building an efficacious vehicle to substantially decrease the spread of the virus. The vaccines formulated had many challenges due to multiple factors such as viral mutations and clinical trial delays. This paper will aim to educate readers on the processes that the vaccine candidates took, and better understand the procedures; additionally, we’ll look at all candidates’ findings that went into clinical trials, assessing, analyzing, and evaluating the 27 vaccine candidates that went into phase III trials and the 13 candidates that went into either phase I/II trials.

## 1. Introduction

Severe Acute Respiratory Syndrome Coronavirus 2 (SARS-CoV-2), better known as Coronavirus (COVID-19), is an infectious disease that is said to have firstly originated in Wuhan, China [1]. The World Health Organization (WHO) first learned of this new virus on 31 December 2019, following the reporting of a cluster of cases of ‘viral pneumonia’ in Wuhan, People’s Republic of China [2]. By the end of January 2020, the WHO officially declared COVID-19 as a pandemic, a Public Health Emergency of International Concern (PHEIC). The virus spread to around 25 countries by early February 2020, the spreading of the virus was due to the lack of action taken in many parts of the world. The spread of the virus since then was rapid and currently, COVID-19 cases are present worldwide in 213 countries, areas, or territories. Hence, guidelines and criteria for diagnosis, treatment, and preventative measures had to be established rapidly due to the increasing number of people that were getting infected with the virus [3]. Researchers worldwide worked and shared their contributions regarding the epidemiology, prevention, treatment, clinical and diagnostic patterns of the COVID-19 virus. Viral detection using RT-PCR identified the SARS-CoV-2 virus to be the disease that has caused this viral transmission worldwide [3].

**Figure 1:**
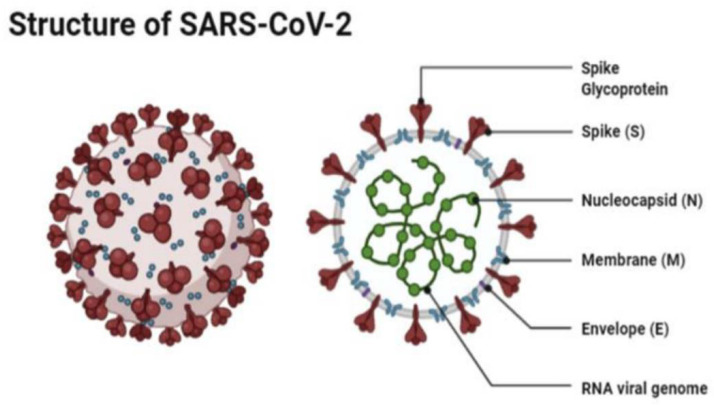
The structure of SAR-CoV-2 under a microscope as illustrated by Agarwal et al. [5].

Severe acute respiratory syndrome coronavirus 2 (SARS-CoV-2) is a beta coronavirus that belongs to the Coronaviridae family. The family is composed of single-stranded positive ribonucleic acid (RNA) viruses [4]. The size of the virus is between 50 and 150 nm in diameter, and its linearity and positive-sense RNA genome are large. SARS-CoV-2 is an enveloped spherical-shaped virus. It has four structural proteins and 16 nonstructural proteins. The structural proteins are the nucleocapsid (N) protein, the membrane (M), the S protein, and the envelope (E) protein. The ribonucleic acid (RNA) is oriented in a 5′-3′direction which makes it a positive sense RNA virus, and the RNA can be read directly as a messenger RNA [4]. The RNA replicase is encoded at the 5′ terminal end. The nonstructural protein 14 (nsp14) has proofreading activity which allows the rate of mutations to stay low. The S protein causes the attachment of the virus to the host cell at the angiotensin-converting enzyme 2 (ACE2) receptor, which is present on the membrane of the host cell. The ACE2 receptors are found in abundance on alveolar cells [4] (Figure 1).

Most infected people will develop mild to moderate respiratory illness and recover without requiring special treatment or hospitalization. Nevertheless, some infected Patients with COVID-19 can develop pneumonia, severe symptoms of acute respiratory distress syndrome (ARDS), and multiple organ failure [6]. Patients with underlying medical conditions such as cardiovascular disease, diabetes, chronic respiratory disease, and cancer, and older people are more likely to develop serious illness. However, Epidemiological studies have shown that mortalities are higher in the elder population and the incidence is much lower in children [7]. COVID-19 affects different people in different ways. Symptoms of COVID-19 consist of two states, the (i) symptomatic state, and the (ii) asymptomatic state. The symptomatic state can be noticed through the patient showing multiple different symptoms such as fever and/or cough [8]. The less common symptoms of COVID-19 include sore throat, headache, aches, pains, diarrhoea, a rash on the skin, discoloration of fingers or toes, and red or irritated eyes. While the more serious symptoms of COVID-19 are difficulty breathing (due to the lowering of oxygen levels) or shortness of breath, loss of speech or mobility, or confusion and chest pain. The transmission of the virus occurs if a person touches a surface contaminated with SARS-CoV-2, and then the hands come into direct contact with mucous membranes such as the eyes, nose, or mouth [9]. Although it is more common that transmission occurs through symptomatic patients, asymptomatic patients who show no symptoms of the virus due to the immune system’s capability of combatting the virus, are the main source of transmission; through their respiratory droplets being airborne, as well as transmitted through virus contaminated containers and foods [10]. Therefore, rapid contact tracing and testing that identifies asymptomatic cases are conducted [11]. Similar to other coronaviruses, SARS-CoV-2 can be established by multiple virus genotypes. This genetic diversity can lead to some advantages for the virus, such as better binding to the receptor, faster replication, and more effective suppression or avoidance of the host’s immune response [12].

### 1.1. COVID-19 Variants

Viruses innately have the ability to mutate constantly and lead to variants. Some variants emerge and disappear while some persist [3]. Mutations to the virus happen during the process of viral replication, that is when the virus attaches to the ACE2 receptor, which is present on the membrane of the host cell. In viral replication and amplification, the assembly of the virions is carried out in the host cell endoplasmic reticulum and Golgi apparatus. During this process, errors can occur in the genome leading to mutations that give rise to variants [3]. A change in the genetic sequence is called a mutation. Mutations can increase the transmissibility and/or virulence of the virus with a possible reduction of vaccine effectiveness [13]. Genomes that differ from each other in genetic sequence are called variants. Variants can differ from each other by one or more mutations. When a phenotypic difference is demonstrated among the variants, they are called strains [3]. So far, COVID-19 has been defined by 17 known mutations (14 non-synonymous mutations and 3 deletions), eight of these mutations have been on the spike protein, the main target site for the vaccination, with at least three of these mutations having a significant biological effect [14]. Several human Coronaviruses (alpha-CoVs, HCoVs-NL63, beta-CoVs, HCoVs-OC43, HCoVs-229E, HCoVs-HKU1, MERS-CoV, SARS-CoV, and ARDS) have been identified [14]. New versions of the Coronavirus Vaccines will appear due to the large genomic potential, rapid mutation capabilities, and high prevalence. The US Department of Health and Human Services (HHS) established the SARS-CoV-2 Interagency Group (SIG) to focus on the rapid characterization of emerging variants and actively monitor their potential impact on critical SARS-CoV-2 countermeasures, such as immunizations, pharmaceuticals, and testing. SARS-CoV-2 variations are divided into four categories under the SIG Variant classification scheme: variants being monitored (VBM), variants of interest (VOI), variants of concern (VOC), and variants of high consequence (VOHC) [15]. The following are some of the variants of coronavirus:

1. 20A/S:439K

This variant The 20A/S:439K variant was initially found in Ireland [16]. It has an S:N439K mutation with the deletions of amino acids at positions 69 and 70 of S proteins that result in an increase in ACE2 binding, resistance to antibodies, and convalescent plasma [16].

2. 20A/S:98F

The 20A/S:98F variant has an S:98F mutation which was found predominantly in Belgium and Netherlands [16].

3. 20C/S:80Y

The 20C/S:80Y variant had 18 nucleotide mutations, possibly related to apolipoprotein B editing complex (APOBEC)-like editing within the host which is found in at least ten countries in Europe [16].

4. 20B/S:626S

The 20B/S:626S variant has an S:626S mutation. This variant is found in 15 countries in Europe and is predominantly seen in Norway, Denmark, and the UK [16].

5. 20B/S:1122L

The 20B/S:1122L variant has an S:V1122L mutation and is found predominantly in Sweden, Norway, and Denmark [16].

6. N440K

Another new variant N440K resulted in the sudden increase in cases in Andhra Pradesh, India. N440K is a new variant with the mutation in the S protein, which has enhanced binding to ACE2 receptors, is 10 to 1000 folds more transmissible, and is resistant to class 3 monoclonal antibodies C135 and REGN10987 [16].

7. 20A.EU1/S:A222V

The 20A.EU1 variant has non-terminal domain (NTD) mutations which do not play a direct role in receptor binding or membrane fusion. This variant was initially identified on 20 June 2020 in Spain but rapidly spread across Europe and many countries [16].

8. 20A.EU2

The 20A.EU2 variant was found in France in June 2020 and has become the second dominant variant in Europe. The notable mutations are S477N, E484K, and N501Y, which demonstrated a slight increase in ACE2 binding, resistance to multiple antibodies, and convalescent sera [16].

9. B.1.526 (20C/S:484K) and B.1.525 (20A/S:484K)

These variants were first identified in New York, USA. The notable mutations are E484K and S477N. While E484K decreases antibody response, S477N increases the attachment process [16].

10. Double Mutant Variant (B.1.617)

This variant is first detected in India. As two mutations are seen in the same virus, this variant is called a “double mutant” variant. There was a significant increase in COVID-19 cases in India. The notable mutations are E484Q and L452R. These variants are at increased risk of transmission and are also resistant to vaccination [16].

11. Triple Mutant Variant (B.1.618)

In addition to E484Q and L425R in double mutant variants, the new triple variant discovered on 20 April 2021, is characterized by the deletion of two amino acids, H146del and Y145del in the S protein [16].

12. US Southern California Variant (CAL.20C)

It was first seen in July 2020 in Southern California and detected again amongst population samples of the same region in October 2020. Its notable mutations are ORF1a: I4205V, ORF1b: D1183Y, S: S13I; W152C and L452R [16].

### 1.2. Vaccines

With the economic, societal, and public health effects of COVID-19, it was essential to develop a vaccine to minimize the severe consequences of this virus. Before the development of the vaccines, some non-pharmaceutical interventions have shown benefits in minimizing the spread of COVID-19. Those non-pharmaceutical interventions included social distancing, wearing of facemasks, and limits of large gatherings. However, they had limited effectiveness due to poor adherence to those practices and unclear advice from ministries of public health. Therefore, the development of an effective COVID-19 vaccine has been a critical need to control the disease and its effects [17].

Global collaboration among pharmaceutical companies, governments, and academic researchers was mounted to develop a COVID-19 vaccine since a publication about the SARS-CoV-2 viral sequence was released on 10 January 2020. The three main authorities that coordinated the vaccine research were World Health Organization, Gavi, and the Coalition for Epidemic Preparedness and Innovation (CEPI). It was reported by CEPI that there were 321 vaccine candidates in development around the globe in September 2020. However, only 40 vaccine candidates progressed to clinical trials in humans in October 2020 and 11 of them were in phase III clinical trials that aimed to provide the safety and efficacy evidence that is required for approval of the vaccine.

The most important outcome that was assessed in these trials is vaccine efficacy. In June 2020, the U.S. Food and Drug Administration (FDA) set a definition for vaccine efficacy and it included two main components. Firstly, for the vaccine to be considered effective it should have the ability to minimize the virus transmission, which means it should prevent the ability of the virus from an infected person to another person. Moreover, the vaccine should have disease-modifying effects in vaccinated individuals. This means that it should decrease the severity of the disease progression and decrease mortality. This definition provided guidance to trial sponsors to set endpoints to their specific settings and population [17]. With this definition, many clinical trials started in several countries and more than 200 million doses of coronavirus vaccines with different vehicles have been delivered [18].

In August 2020, the government of Russia announced its creation of a vaccine, named Sputnik V. While the Chinese biotech company, Sinovac Biotech Ltd., launched clinical trials of an inactivated virus vaccine, named CoronaVac between April and July 2020. Around the same time, clinical trials of two novel vaccines (made by biotech companies Moderna and Pfizer-BioNTech) began in the United States. In December 2020, the United States Food and Drug Administration authorized two mRNA vaccines for emergency use. In February 2021, a viral vector vaccine made by Johnson & Johnson Janssen was also authorized for emergency use [19].

### 1.3. Vaccine Vehicles

#### 1.3.1. Traditional Whole-Pathogen Vaccines

COVID-19 vaccines have a variety of compositions, some are traditional whole-pathogen vaccines and others are new-generation vaccines that work by new mechanisms. The traditional whole-pathogen vaccines include two types. Firstly, the live–attenuated vaccines, which are live pathogens but with reduced virulence. This type of vaccine introduced an infection similar to the real infection which triggers an immune response and thus an immunological memory that will fight against future infection. However, live-attenuated vaccines have potential safety concerns because they have higher reactogenicity and they can cause infection in immunocompromised patients. In contrast, the second type of traditional whole-pathogen vaccines are considered safer. The inactivated vaccines consist of chemically or thermally inactivated viruses. They are considered to be safer because live pathogens are not involved. However, in terms of efficacy, they result in lower immunogenicity and might require multiple dosing [20].

#### 1.3.2. The New-Generation Vaccines

Due to the safety concerns associated with the traditional vaccines, a new generation of vaccines was developed that only incorporate the antigens that are responsible for pathogenesis instead of the whole pathogen. However, it requires more time and effort to study and understand the exact pathogenesis of the virus compared to injecting it as a whole.

Fortunately, the COVID-19 virus is very similar to SARS-CoV and the Middle East Respiratory Syndrome Coronavirus (MERS-CoV), which has been well studied and investigated for many years.

The new generation of COVID-19 vaccines can be classified based on the carrier of the antigen into Recombinant protein vaccines, viral vector-based vaccines, bacterial vector-based vaccines, plasmid DNA vaccines, Messenger RNA vaccines, and trained immunity-based vaccines [20].

#### 1.3.3. Recombinant Protein Vaccines

These vaccines use a protein-fragment-like Receptor Binding Domain (RBD) or complex of RBD with a carrier protein as an antigen. Then, the antigen presenting cells (APC) will engulf the antigen, and it will be digested in the endosome. A very small fraction of the ingested antigen will be expressed on the cell to respond to the major histocompatibility complex (MHC) II molecules, which initiates an immune response. Moreover, it was shown that recombinant protein SARS-CoV vaccines injected into animal models resulted in the production of neutralizing antibodies.

However, recombinant protein vaccines require an adjuvant in the formulation such as Matrix-M because by itself it only triggers a specific humoral response and only induces partial protection against the infection [20].

#### 1.3.4. Viral Vector-Based Vaccines

The viral vector vaccines resemble the viral infection disease state. It is a modified version of the virus, which is not the virus that causes the disease, known as a vector virus. This modified virus is harmless [21]. They can produce stronger immune responses compared to recombinant protein vaccines. Viral vector vaccines work by cloning the antigen into a viral vector that is unable to reproduce. Lentivirus, adenovirus, and adeno-associated virus (AAV) is the most commonly used vectors. One of the vectors that were used in the past for SARS-CoV vaccine candidates was the AAV vector.

#### 1.3.5. Bacterial Vector-Based Vaccines

They are considered another way for developing vector-based vaccines. The most common example is the non-pathogenic lactic acid bacteria (LAB). It is implemented in Symvivo’s COVID-19 vaccine candidate which is currently in clinical trials. Bacterial vector-based vaccines have some advantages including low cost of manufacturing and better stability as they can be prepared by lyophilization [20].

#### 1.3.6. Plasmid DNA Vaccines

Plasmids are circular deoxyribonucleic acid (DNA) vectors that can be used as vaccines to prevent various types of diseases [22]. Plasmid DNA vaccines have a better safety profile because they do not use a live virus. Moreover, the double-strand DNA molecules are more stable than m RNA, virus, and protein. In addition, it is suitable for long-term storage as it can be freeze-dried. This vaccine works by injecting it along with electrodes. After that, an electrical pulse is applied to allow the opening of the cell membrane and hence the entry of the plasmid into the cell.

#### 1.3.7. Trained Immunity-Based Vaccines

Trained immunity-based vaccines are non-infectious vaccines, they differ from other conventional vaccines by stimulating the innate immunity that provides protection against unrelated pathogens. While other conventional vaccines target the adaptive immunity that provides pathogen-specific protection. Trained immunity-based vaccines are conceived to confer a broad protection far beyond the antigens they contain [23]. There is a current clinical trial that investigates the ability of the Bacille Calmette- Guerin (BCG) vaccine which was developed for tuberculosis disease to be effective against COVID-19 [20].

#### 1.3.8. Messenger RNA Vaccines

**Figure 2:**
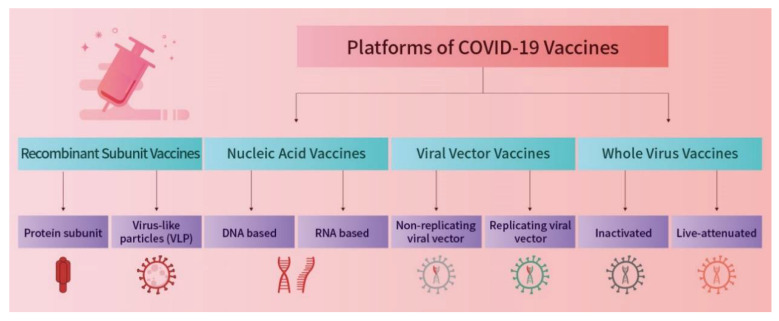
**A** schematic diagram showing the different vehicles used to combat the COVID-19 virus. [26].

These are the newest generation of vaccines and all their components can be prepared by chemical synthesis. mRNA vaccines teach our cells how to make a protein that will trigger an immune response inside our bodies [24]. They work by introducing mRNA as an intermediate to be translated to an antigen to induce an immune response in the host. There are many advantages to using mRNA vaccines. Firstly, they are easily manufactured, and they do not require a lot of time because RNA synthesis can be conducted once the sequence encoding the immunogen is available. Moreover, they are considered to have a better biosafety profile compared to DNA-based vaccines because the antigen translation happens in the cytoplasm rather than the nucleus and because it carries a short sequence to be translated so it does not interact with the host genome [25]. The different vaccine vehicles are illustrated in Figure 2.

Trials on vaccines: different phases in clinical trials that the vaccines went through:

For the development of the COVID-19 vaccine, the vaccine must undergo three clinical phases.

### 1.4. Phase I Clinical Trials

This is the first stage where the vaccine is administered to humans [27]. In phase I clinical trials, dozens or hundreds of healthy adult volunteers are enrolled to assess the initial safety profile of the vaccine and to compare different vaccine doses. If the vaccine shows an acceptable safety profile from phase I, it will proceed to phase II clinical trials.

### 1.5. Phase II Clinical Trials

In Phase II vaccine trial, a larger group of several hundred individuals participate in testing [28]. Phase II clinical trials aim to continue to assess the safety of the vaccine, test the immune response to the vaccine in healthy people compared to those who remained unvaccinated. In many trials, they combine both phase I and phase II into phase I and II clinical trials where they achieve the aims of both phases together.

If the results from the phase II clinical trials are promising, then the vaccine will continue to phase III clinical trials.

### 1.6. Phase III Clinical Trials

All of the current phase III trials are designed as individually randomized, placebo-controlled clinical trials (RCTs). These trials will help ensure that necessary data are generated as quickly and efficiently as possible while maintaining high ethical and scientific standards [29]. Phase III clinical trials are conducted on thousands or sometimes more than 100,000 volunteers who will be randomly assigned to be vaccinated or remain unvaccinated. This allows the researchers to determine whether the vaccine will provide protection against the virus in those who received the vaccine compared to unvaccinated subjects. Therefore, phase III clinical trials are very important to provide strong evidence about how safe and protective the vaccine candidate is. Sometimes researchers can combine phase II and phase III clinical trials into phase II/III clinical trials that achieve the aims of both trials.

After that, if the results from phase I, II, and III clinical trials showed that the vaccine candidate is safe and effective, national authorities and regulatory agencies will review this evidence and decide whether the vaccine candidate should be approved and authorized.

However, after phase I, II, and III trials, and after authorization, the vaccine candidate continues to be monitored to ensure their safety and effectiveness through phase IV.

### 1.7. COVID-19 Vaccines Developed More Rapidly Than Ever Before

In the past, the process of developing a vaccine took years to provide enough evidence about its safety and efficacy. This slow progress was due to a lack of sufficient funding, the small number of researchers working on vaccine development, and insufficient resources.

However, the development of COVID-19 vaccines was accelerated without delay due to their urgency and rapid spread [30]. The accelerated developments resulted from devoting huge funding, and many resources and investigating multiple candidates by many countries and companies [31].

This literature review aims to analyze all the vaccine candidates that went to phase II and above with the rationale behind the completion of some vaccines to phase III and why others failed to continue to phase III. Moreover, this paper will provide comprehensive information about COVID-19 infection, its symptoms, the structure of the virus, the variants that we have become aware of, and how they differ. Furthermore, it will inform the literature about vaccines developed for COVID-19 infection, vaccine vehicles, and trials on vaccines. This paper shall open doors for further research and a better understanding of the reasons that hinder vaccines from reaching the market, which is important to be taken into consideration when developing new vaccines for viruses in the future.

## 2. Results

Many vaccine candidates were able to proceed from phases I and II to phase III clinical trials. Those candidates are summarized in Table 1 with their developer, country of origin, the technology used to develop them, clinical trials they went through, and the findings of the completed phases of those trials. For vaccines that have findings for phase III clinical trials, earlier findings of Phases I and II are discussed below in the table.

**Clinical phase I and phase II findings for vaccines that have completed Phase III findings:**

- **Janssen:** Phase I-IIa findings include: enough antibodies to neutralize the virus, Injection site reactions: pain, and redness of the skin. General side effects: headache, fatigue, myalgia, nausea, and fever [36].
- **Sinopharm BIBP:** In this phase I/II trial, the BBIBP-CorV inactivated vaccine, given as a two-dose immunisation, was safe and well tolerated at all three doses in both age groups. A robust humoral immune response was observed in 100% of vaccine recipients and the most common adverse effects were pain and fever [35].
- **CoronaVac:** Two doses of CoronaVac at different concentrations and using different dosing schedules were well tolerated and moderately immunogenic in healthy adults aged 18–59 years. The incidence of adverse reactions in the 3 μg and 6 μg groups were similar, indicating no dose-related safety concerns but more long-term follow-up is needed. Furthermore, most adverse reactions were mild, with the most common symptom being injection-site pain. All in all, CoronaVac was well tolerated and induced humoral responses against SARS-CoV-2 (neutralizing antibodies), which supported the approval of emergency use of CoronaVac in China, and three phase III clinical trials that are ongoing in Brazil (NCT04456595), Indonesia (NCT04508075), and Turkey (NCT04582344) [39].
- **Novavax:** After phase I/II trials, the adverse effects produced were null or mild and of short duration. The addition of adjuvant enhanced the immune responses elicited by the vaccine candidate and resulted in cellular responses that exhibited a Th1-skewed phenotype. Anti-S IgG and neutralizing antibodies induced by vaccination exceeded those detected in convalescent sera from COVID-19 patients. All phase III clinical trials are still ongoing; therefore, their results have not been reported [44].
- **Covaxin:** The interim findings from the phase I clinical trial, the vaccine was well tolerated in all dose groups with no vaccine-related serious adverse events. Both humoral and cell-mediated responses were observed in the recipients of the Algel-IMDG-based vaccines. The most common adverse event was pain at the injection site, followed by headache, fatigue, and fever. The overall incidence of solicited local and systemic adverse events in this study was 14–21% in all vaccine-treated groups, which is noticeably lower than the rates for other SARS-CoV-2 vaccine platform candidates 18, 19, 20, 21, 22, 23. BBV152 induced binding and neutralising antibody responses that were similar to those induced by other SARS-CoV-2 inactivated vaccine candidates [45]. In the phase II trial, BBV152 showed better reactogenicity and safety outcomes, and enhanced humoral and cell-mediated immune responses compared with the phase I trial [46]. The 6 μg with Algel-IMDG formulation has been selected for the phase III efficacy trial. However, the refusal rate for Phase III trials was much higher than that for Phase I and Phase II. As a result, only 13,000 volunteers had been recruited by 22 December with the number increasing to 23,000 by 5 January [48].
- **Sputnik light:** In terms of safety outcomes, the “Sputnik Light” vaccine was well tolerated both in seronegative and seropositive groups (appendix p20). The most common solicited systemic adverse effect was flu-like syndrome equally found in seronegative (47/96 [49·0%]) and seropositive (7/14 [50·0%]) groups. Interestingly that only participants without immunity to SARS-CoV-2 complained of muscle and joint pain after vaccination (5/96 [5·2%]). Moreover, “Sputnik Light” showed to be immunogenic, inducing both binding and neutralizing antibody responses in 100% (93/93) and 81·7% (76/93) of seronegative participants by day 42, respectively. These results from phase I and II have made a basis for provisional vaccine approval for clinical use issued on 6 May 2021 (registration of LP-006993). The Provisional licensure made it possible to start an international multicenter randomized, double-blind, placebo-controlled phase III clinical trial (NCT04741061) to evaluate the efficacy, immunogenicity, and safety of the “Sputnik Light” vector vaccine in the parallel assignment of the subjects in prophylactic treatment for SARS-CoV-2 infection [35].
- **Convidecia:** Phase II results showed that the vaccine induced seroconversion of the neutralising antibodies in 59% and 47% of participants, and seroconversion of binding antibodies in 96% and 97% of participants. Positive specific T-cell responses measured by IFNγ-ELISpot were found in 90% and 88% of participants receiving the vaccine. Moreover, most reactions reported post-vaccination were mild or moderate. Although the proportions of participants who had adverse reactions such as fever, fatigue, and injection site pain were significantly higher in vaccine recipients than those in placebo recipients, adverse reactions within 28 days were generally not severe and resolved within a short period of time [53]. In addition, phase III clinical trial has shown the efficacy of Convidecia, however, the efficacy dropped from 68.83% after two weeks to 65.28% after four weeks. Hence, a booster shot may be required to develop desired neutralizing antibodies and the efficacy might also increase to 90% [55].
- **Sinopharm WIBP:** In this is the first report of phase I and II clinical trials of a whole-virus-inactivated COVID-19 vaccine among healthy adults. The inactivated vaccine was well tolerated in all dose groups under different injection procedures with no vaccine-related serious adverse events. The most common adverse reaction was injection site pain, which was mild and self-limiting. The incidence rate of adverse reactions in the current study (15.0% among all participants) was lower compared with the results of other candidate vaccines. Moreover, The neutralizing antibody response was monitored over 14 days after injections in the current preliminary report, and the results suggested that the inactivated vaccine may effectively induce antibody production. The results in both phases indicated that a longer interval (21 days and 28 days) between the first and second injections produced higher antibody responses compared with a shorter interval schedule (14-day group) [57].
- **Abdala (CIGB-66):** The product was well tolerated. Severe adverse events were not reported. Adverse reactions were minimal, mostly mild and local (from the injection site) and resolved in the first 24–48 h without medication. In phase I, at day 56 seroconversion of anti-RBD IgG was seen in 95.2 % of the participants (20/21) for the 50 μg group whereas neutralizing antibodies to SARS-CoV-2 were seen in 80 % of the participants (8/10) for the 50 μg group. In phase II, at day 56 seroconversion of anti-RBD IgG was seen in 89.2% of the participants (214/240) for the 50 μg group whereas neutralizing antibodies to SARS-CoV-2 were seen in 97.3% of the participants (146/150) for the 50 μg group [62].
- **EpiVacCorona:** Phase I–II clinical trials showed that all local reactions in response to vaccine administration were mild, such as short-term pain at the injection site. There were no signs of the development of local or systemic adverse reactions. The two-dose vaccination scheme induced the production of antibodies, specific to the antigens that make up the vaccine, in 100% of the volunteers. Seroconversion with a neutralizing antibody titer ≥ 1:20 was reported in 100% of the volunteers 21 days following the second immunization dose which means that the peptide-based EpiVacCorona Vaccine has low reactogenicity and is a safe, immunogenic product [65].
- **ZF2001:** According to phase I and II clinical trials, most of the local and systemic reactogenicity was mild or moderate (grade 1 or 2 adverse events). The most common solicited local adverse events were injection-site pain, redness, and itch. The most common solicited systemic adverse events were cough, fever, and headache but no vaccine-related serious adverse events were reported. Moreover, neutralizing antibodies were detected in the serum of vaccinated participants. Consequently, the protein subunit vaccine ZF2001 appears to be well tolerated and immunogenic. The safety and immunogenicity data from the phase I and II trials support the use of the 25 μg dose in a three-dose schedule in the phase III trial for large-scale evaluation of ZF2001’s safety and efficacy [70].
- **Soberana 02:** Based on phase I and phase II clinical trials, the most frequent adverse event was local pain with no serious related AEs reported. Phase IIa confirmed the safety results in 60–80-year-old subjects. In phase-I SOBERANA 02–25 mg elicited a higher immune response than SOBERANA 02–15 mg; in consequence, the higher dose progressed to phase IIa. Phase IIa results confirmed the immunogenicity of SOBERANA 02–25 mg even in the 60–80 age range. Two doses of SOBERANA02-25 mg elicited an immune response (neutralizing antibodies and specific T cell response) similar to that of the Cuban Convalescent Serum Panel; it was higher after both the homologous and heterologous third doses; the heterologous scheme showing a higher immunological response [75].
- **BNT162b2:** Based on phase I and phase II clinical trials, the immune system was found to generate neutralizing antibody response peaking 7 days after a booster dose. side effects included pain at the injection site, fatigue, headache, chills, muscle pains, joint pain, and fever [79].
- **QazCovid-in commercially known as QazVac:** In the phase I trial, there was a 59% fourfold increase of antibody titres in MNA after one vaccine dose and amounted to 100% after two doses. Neutralizing antibody titres reached the geometric mean titre (GMT) of 100 after the administration of two doses [80]. A statistically significant increase in the levels of pro-inflammatory cytokines after vaccination indicated the Th1-biased response. On day 180, 40% of placebo-treated subjects demonstrated a statistically significant increase in the levels of antibodies measured by both ELISA and MNA, which suggests the infection with SARS-CoV-2. In the phase II trial, 100% of subjects aged 18–49 years seroconverted for SARS-CoV-2 on day 21 after the first dose, yielding the GMTs of 32 or 30 in the one- and two-dose groups. Amongst ≥50-year-old subjects, the number of seroconversions in the two-and one-dose groups on day 21 was 94% and 92% with the respective GMTs of 25 and 24. After the second dose, the seroconversion rate reached 100%; however, the GMT was significantly lower when compared with the corresponding value measured in subjects aged 18–49 years (83 vs. 143). In both trials, specific antibodies were detected in MNA and ELISA on study day 180, but the titres dropped in comparison to day 42 [80].
- **Chinese Academy of Medical Sciences COVID-19 vaccine:** Based on phases I and II clinical trials, on the 28th day after immunization with different doses of vaccine by 0,14 and 0,28 procedures, the effective neutralizing antibody seroconversion reached more than 94% in different age groups [81,82].
- **Zycov-D:** Phase I trial found the vaccine to be “safe, well-tolerated and immunogenic”. 12/48 (25%) subjects reported at least one AE during the study. There were no deaths or serious adverse events reported [83].
- Sinopharm CNBG: The two inactivated Chinese vaccines showed efficacy rates of 72.8 percent and 78.1 percent, respectively, against symptomatic COVID-19 cases, with rare serious adverse effects reported, according to an interim analysis of the ongoing trials [86].
- **Corbevax:** A previous Phase I/II clinical trial evaluated the safety and immunogenicity of the vaccine candidate in about 360 healthy subjects aged 18–65 years. The schedule consisted of two doses for each participant, administered via intramuscular injection, 28 days apart. Bio E’s novel COVID-19 vaccine was safe, well-tolerated, and immunogenic [87].

Vaccine candidates can also go through the first phase of clinical trials. However, if there were questions about the safety or the efficacy of the vaccine, the trials may be paused or abandoned. Regarding the safety of the vaccine candidate, if the investigators observe worrying symptoms in the volunteers, they can pause the trial. While the trials may also be abandoned if they indicate that the vaccine candidate is not effective against COVID-19. There are about 13 vaccine candidates that were abandoned to continue the clinical trials summarized in Table 2.

**The reasons behind the pause of the clinical trials of vaccine candidates are summarized in Table 2**.

Each of these vaccines had different reasons for being abandoned. For example, GX-19N needs improvement to be competitive with other vaccines as suggested by results from the Phase I/II trial [88,89].

While the Imperial College London found that the self-amplifying RNA platform was safe and promising, the vaccine (COVAC1) couldn’t generate a promising immune response [89]. Additionally, Sanofi decided to pull the plug on its own mRNA COVID-19 vaccine (MRT5500) program, when Pfizer-BioNTech and Moderna vaccines became widely available [88,91].

- On 27 January 2021, **OncoSec Immunotherapies** began dosing participants in its Phase I trial to test the safety of CORVax12. In November, a spokeswoman said that OncoSec was no longer investigating the vaccine [88,92]
- **Merck** acquired the Austrian firm **Themis Bioscience** in June 2020 to develop their vaccine V591, which had been originally developed at **Institut Pasteur**. However, on 25 January 2021, Merck announced it was abandoning the effort because the vaccine provoked a response that was weaker than a natural infection. In addition to its project with Themis, **Merck** partnered with **IAVI** on a second viral vector vaccine V590. However, on 25 January 2021, they announced they were abandoning the effort because the vaccine failed to trigger an immune system comparable to what happens in a natural infection of COVID-19 [88,93]
- AdCOVID vaccine was developed to be a nasal spray vaccine for COVID-19, delivering the Ad5 adenovirus to the airway because it was suggested that a nasal spray could be more effective for blocking the transmission of the virus than vaccines given by injection. However, on 29 June 2021, Altimmune decided to abandon their COVID-19 vaccine because they found that sprays of the vaccine produced lower levels of antibodies than other authorized COVID-19 vaccines [88,94].
- IVX-411 vaccine was stopped at phase1/2 trial on 25 March 2022 because it did not deliver a stronger immune response than natural infection [88,95].
- CoVepiT received approval to go through phase I trial, however on 19 July, OSE decided to voluntarily pause its trial after the development of some adverse drug reactions to the vaccine. Moreover, OSE decided to pause its development because some therapeutics and vaccines have already proven successful in patients [88,96].
- QazCoVac-P is the second vaccine developed by the Research Institute for Biological Safety Problems. It was developed as a protein subunit vaccine unlike their first vaccine, QazVac, which was made from inactivated viruses. Kazakhstani researchers started a phase I/II trial on 15 June 2021. However, there was no evidence of QazCoVac-P use in Kazakhstan until February 2022. Therefore, on 28 April, Kazakhstani health officials decided that is unnecessary to continue the production of QazCoVac-P because there were enough QazVac doses to cover the population [88].
- NBP2001 won approval on 23 November 2020 to go to phase I trial. In the trial’s registry, it was scheduled to end the trial on April 2021, however, on SK BioScience’s website the company said that the trial is complete and they did not launch a phase II trial to carry the research forward. Additionally, the company was busy pushing another vaccine, called GBP510 through a phase III trial [88].
- V451 vaccine was developed by Queensland University. In July, the university launched a phase I trial, combining coronavirus spike proteins with an adjuvant made by CSL. While the Phase I trial safety and immunogenicity data are positive, the researchers found that volunteers were getting false positive tests for HIV even though they were not actually infected with the virus. In February 2021, the researchers reported that these false positive results were due to the way the researchers developed the vaccine. However, the Australian government decided to stop the trial because that false positive HIV results would lead to hesitancy in getting that vaccine [88,97].
- Finally, Fakhravac was launched on 16 March 2021 in Iran. It completed phase I and then entered phase II in June. In September 2021, it gained emergency use authorization. However, in October Iran decided to abandon its production of it as Iranians turned to imported vaccines instead [88,98].

## 3. Conclusions

Vaccines save millions of lives every year, and the formulation of a safe and effective vaccine for COVID-19 has allowed people to return to somewhat of normality. After almost three years of the pandemic, COVID-19 vaccinations have effectively and significantly reduced the course of the pandemic, saving many lives globally. Based on official reports, the vaccinations could have saved up to 20 million deaths worldwide. This study looked at 40 vaccine candidates that went into clinical trials, discussing and evaluating their platforms, trials, findings, and limitations for each of the candidates. It was seen that most candidates that went into phase III clinical trials, had an effective profile of between 65 to 95%, hence reducing the incidence and severity of SARS-CoV-2 infection. This research paper also showed that all candidates that have been approved by the WHO are safe and effective for use, without compromising on any standards whilst assessing their capability to combat the virus. This review aimed to disseminate all the information that has been achieved in clinical trials and be collated into one article. The objective of this review paper was to put all the data onto one paper, as this is not currently available, and hence make it easier to read all information from one source, and we, the authors, believe this was achieved via this paper.

## Figures and Tables

**Table 1:** Vaccines that went to phase III.

Vaccine Candidate	Developer	Country	Technology Used	Phase I Study	Phase II Study	Phase III Study	Completed Phases Findings	Clinical Trials Sites
mRNA-1273 [32,33]	Moderna	United States of America	Lipid nanoparticle dispersion containing mRNA	Phase I trial to study Short term efficacy and systemic adverse events.	Phase II trial to assess the safety, reactogenicity, and immunogenicity.	Phase III (30,420) Interventional; randomized, placebo-controlled study for efficacy, safety, and immunogenicity	Phase I (45) Antibody response was detected after the administration of two doses; side effects included fever, fatigue, headache, muscle ache, and pain at the injection site	89 sites in the USA
Sputnik V [34]	Gamaleya Research Institute of Epidemiology and Microbiology	Russia	Adenovirus vector vaccine (recombinant adenovirus type 5 and 26 vectors)	An open label, prospective, non-randomized combined phase I/II trial to evaluate the safety, reactogenicity, and immunogenicity.	An open label, prospective, non-randomized combined phase I/II trial to evaluate the safety, reactogenicity, and immunogenicity.	Phase III (40,000) Randomized double-blind, placebo-controlled to evaluate efficacy, immunogenicity, and safety	Phase I–II (76) Neutralizing antibody and T cell responses	28 sites in Europe, South America, and Asia
Ad26.COV2.S [35,36]	Janssen Pharmaceutica (a subsidiary of Johnson & Johnson)	United States of America	viral vector vaccine	Phase I trial established the safety, reactogenicity, and immunogenicity of Ad26.COV2.S	Phase II a trial established the safety, reactogenicity, and immunogenicity.	A Randomized, Double-blind, Placebo-controlled Phase III Study to Assess the Efficacy and Safety of Ad26.COV2.S for the Prevention of SARS-CoV-2-mediated COVID-19 in Adults Aged 18 Years and Older	Phase III findings: The vaccine showed efficacy against severe/ critical COVID-19, hospitalization, and COVID related deaths.	16 trials in 18 countries
Sinopharm BIBP [37,38]	Sinopharm Beijing Institute of Biological Products	China	Inactivated vaccine	randomised, double-blind, placebo-controlled, phase I/II trial to assess safety, tolerability, and immunogenicity	randomised, double-blind, placebo-controlled, phase I/II trial to assess safety, tolerability, and immunogenicity	Randomized, Double-Blind, Placebo Parallel-controlled Phase III Clinical Trial to Evaluate the Efficacy, Immunogenicity, and Safety of the Inactivated SARS-CoV-2 Vaccine (Vero Cell) in an Argentine Healthy Population Aged Between 18 and 85 Years	Phase III findings, efficacy against severe COVID and hospitalization.	19 trials in 10 countries
CoronaVac [39,40,41]	Sinovac Biotech	China	Inactivated virus	A Randomized, Double-Blinded, Placebo-Controlled, Phase Ⅰ/Ⅱ Clinical Trial, to Evaluate the Safety, tolerability, and Immunogenicity of the SARS-CoV-2 Inactivated Vaccine in Healthy Adults Aged 18~59 Years	A Randomized, Double-Blinded, Placebo-Controlled, Phase Ⅰ/Ⅱ Clinical Trial, to Evaluate the Safety, tolerability, and Immunogenicity of the SARS-CoV-2 Inactivated Vaccine in Healthy Adults Aged 18~59 Years	Phase III, randomized, multicenter, endpoint-driven, double-blind, placebo-controlled clinical trial to assess the efficacy and safety of the adsorbed vaccine COVID-19 (inactivated) produced by Sinovac.	Phase III results from Brazil showed efficacy against symptomatic infections, cases that require medical treatment, and against severe, hospitalized, and fatal cases.	28 trials in 8 countries. China, Turkey, Brazil, Indonesia, Chile, Philippines, Thailand, and Hong Kong.
Novavax (NVX-CoV2373) [42,43,44]	Novavax	Australia	Protein subunit: nanoparticle technology which contains the full-length SARS-CoV-2 spike protein and a Matrix-M1 adjuvant	A Phase I/II, Randomized, Observer-Blinded Study to Evaluate the Safety and Immunogenicity of a Quadrivalent Hemagglutinin Nanoparticle Influenza and SARS-CoV-2 rS Nanoparticle Combination Vaccine With Matrix M1™ Adjuvant	A Phase I/II, Randomized, Observer-Blinded Study to Evaluate the Safety and Immunogenicity of a Quadrivalent Hemagglutinin Nanoparticle Influenza and SARS-CoV-2 rS Nanoparticle Combination Vaccine With Matrix M1™ Adjuvant	randomized, placebo-controlled, observer-blinded study to evaluate the efficacy, safety, and immunogenicity of NVX-CoV2373 with Matrix-M in up to 10,000 subjects aged 18 to 84 years	High titers of neutralizing antibodies in all participants in phase I/II CD4+ T cell activation in all participants in phase I/II	11 trials in 7 countries including Australia, the US, the UK, Northern Ireland, Mexico, India, and Puerto Rico.
Covaxin [45,46,47,48]	Bharat Biotech	India	Inactivated virus	An Adaptive Phase I, Followed by Phase II Randomized, Double-blind, Multicenter Study to Evaluate the Safety, Reactogenicity, Tolerability, and Immunogenicity of BBV152 in Healthy Volunteers who receive two intramuscular doses of BBV152 vaccine formulations or Placebo	An Adaptive Phase I, Followed by Phase II Randomized, Double-blind, Multicenter Study to Evaluate the Safety, Reactogenicity, Tolerability, and Immunogenicity of BBV152 in two arms of healthy volunteers who will receive two intramuscular doses of BBV152 vaccine formulations (BBV152-A & BBV152-B) in a 1:1 ratio with dosage schedule on Day 0 and Day 28.	An Event-Driven, Phase III, Randomized, Double-blind, Placebo-controlled, Multicenter Study to Evaluate Efficacy, Safety, Immunogenicity, Lot-to-Lot Consistency of BBV152, and a Whole-Virion Inactivated SARS-CoV-2 Vaccine in Adults ≥ 18 Yrs of Age	In the phase II trial, BBV152 with a dose of 6 μg showed better reactogenicity and safety outcomes and enhanced humoral and cell-mediated immune responses compared with the phase I trial (the dose was 3 μg)	7 trials in India
Sputnik light [49,50,51]	Gamaleya Research Institute of Epidemiology and Microbiology.	Russia	Viral vector	An Open Study on the Safety, Tolerability, and Immunogenicity of “Sputnik Light” t Vaccine for Prevention of Coronavirus Infection Caused by the SARS-CoV-2 Virus	Randomised Phase II Study to Assess the Immunogenicity and Safety of Heterologous SARS-CoV-2 Vaccine Schedules (rAd26-rAd5, rAd26-rAd26, rAd26-ChAdOx1 and rAd26-mRNA-1273).	A Phase III, Randomized, Double-blind, Placebo-controlled International Multicenter Study to Evaluate Efficacy, Immunogenicity and Safety of the Sputnik-Light Vector Vaccine in the Parallel Assignment of the Subjects in Prophylactic Treatment for SARS-CoV-2 Infection	According to phase III results, a single shot of the Sputnik light vaccine has shown efficacy against severe COVID and the single dose regimen solves the challenge of immunizing large groups in a shorter time to achieve herd immunity faster.	4 trials in 2 countries: Russia and Argentina.
AD5-nCOV, (Convidecia) [52,53,54,55]	CanSino Biologics	China	Viral vector vaccine	Randomized, double-blind, placebo-controlled I/II clinical trial, in order to evaluate the safety and immunogenicity of Recombinant Novel Coronavirus Vaccine (Adenovirus Type 5 Vector) for Inhalation in adults 18 years of Age and Older.	A Randomized, Double-blind, Placebo-controlled Phase II Clinical Trial to Evaluate the Safety and Immunogenicity of the Recombinant Novel Coronavirus Vaccine (Adenovirus Vector) in Healthy Adults Aged Above 18 Years	A Global Multicenter, Randomized, Double-blind, Placebo-Controlled, Adaptive Designed Phase Ⅲ Clinical Trial to Evaluate the Efficacy, Safety, and Immunogenicity of Ad5-nCoV in Adults 18 Years of Age and Older	Data from the late-stage clinical trials, single-dose vaccine, CanSino, is 65.7% effective in preventing symptomatic and 90.98% effective in preventing severe COVID-19 infection. However, the efficacy dropped from 68.83% after two weeks to 65.28% after four weeks. Hence, a booster shot may be required to develop desired neutralizing antibodies and the efficacy might also increase to 90%	12 trials in 6 countries including China, Chile, Mexico, Russia, Argentina, and Pakistan.
Sinopharm WIBP [56,57,58,59]	Sinopharm	China	Inactivated virus	Evaluation of the safety and immunogenicity of inactivated Novel Coronavirus Pneumonia (COVID-19) vaccine (Vero cells) in a healthy population aged 6 years and above in a randomized, double-blind, placebo parallel-controlled phase I/II clinical trial	Evaluation of the safety and immunogenicity of inactivated Novel Coronavirus Pneumonia (COVID-19) vaccine (Vero cells) in a healthy population aged 6 years and above in a randomized, double-blind, placebo parallel-controlled phase I/II clinical trial	Multicenterouble Blind, Parallel Placebo-Controlled, Phase III Clinical Trial to Evaluate the Protective Efficacy, Safety, and Immunogenicity of Inactivated SARS-CoV-2 Vaccines (Vero Cell) in a Healthy Population Aged 18 Years Old and Above	In this phase III randomized trial in adults, 2 whole-in phase III trial, virus inactivated vaccine showed an efficacy of 72.8% against symptomatic COVID-19 cases and it had rare serious adverse events.	8 trials in 7 countries including China, Egypt, Morocco, UAE, Bahrain, and Jordan
Abdala (CIGB-66) [60,61,62,63]	Center for Genetic Engineering and Biotechnology	Cuba	Protein subunit vaccine	Randomized controlled trial double-blind, placebo, factorial, phase I/II to evaluate the safety and immunogenicity of the vaccine candidate CIGB-66 against SARS-CoV-2	Randomized controlled trial double-blind, placebo, factorial, phase I/II to evaluate the safety and immunogenicity of the vaccine candidate CIGB-66 against SARS-CoV-2	Phase III, a multicenter, randomized, double-blind, placebo-controlled clinical trial for the evaluation in adults of the efficacy, safety, and immunogenicity of the vaccine candidate CIGB-66 against SARS-CoV-2. (COVID-19)	Late-phase clinical trial data revealed that Abdala is 92.28% effective after the full, three-dose cycle.	5 trials in 1 country: Cuba
EpiVacCorona [64,65,66,67]	VECTOR center of Virology	Russia	Peptide subunit	Simple, Blind, Placebo-controlled, Randomized Study of the Safety, Reactogenicity, and Immunogenicity of Vaccine Based on Peptide Antigens for the Prevention of COVID-19 (EpiVacCorona), in Volunteers Aged 18–60 Years (I–II Phase)	Simple, Blind, Placebo-controlled, Randomized Study of the Safety, Reactogenicity, and Immunogenicity of Vaccine Based on Peptide Antigens for the Prevention of COVID-19 (EpiVacCorona), in Volunteers Aged 18–60 Years (I–II Phase)	Multicenter Double-blind Placebo-controlled Comparative Randomized Study of the Tolerability, Safety, Immunogenicity and Prophylactic Efficacy of the EpiVacCorona Peptide Antigen-based Vaccine for the Prevention of COVID-19, With the Participation of 3000 Volunteers Aged 18 Years and Above (Phase III–IV)	Phase III trials showed that EpiVacCorona provokes an immune reaction against COVID-19 and promotes the further development of immunity with 100% efficacy and a very low side effects profile.	3 trials in 1 country (Russia)
ZF2001 (Zifivax) [67,68,69,70,71,72]	Anhui Zhifei Longcom	China	Protein subunit	A Multi-center, Double-blind, Randomized, Placebo Parallel Controlled, Safety and Tolerability Phase I Clinical Trial of Recombinant Novel Coronavirus Vaccine (CHO Cells) in Healthy People Between 18 and 59 Years of Age	A Randomized, Blinded, Placebo-controlled Trial to Evaluate the Immunogenicity and Safety of a Recombinant New Coronavirus Vaccine (CHO Cell) With Different Doses and Different Immunization Procedures in Healthy People Aged 18 to 59 Years	A Phase III Randomized, Double-blind, Placebo-controlled Clinical Trial in 18 Years of Age and Above to Determine the Safety and Efficacy of ZF2001, a Recombinant Novel Coronavirus Vaccine (CHO Cell) for the Prevention of COVID-19	The latest data from phase III clinical trials showed that the efficacies of the ZF2001 vaccine were 77.54% and 92.93% against Delta and Alpha infections, respectively. In addition, the efficacies of all cases of COVID-19 (81.76%) and severe forms (100%) were also high.	11 trials in 5 countries including China, Ecuador, Indonesia, Pakistan, and Uzbekistan.
Soberana 02 [73,74,75,76,77]	Finlay Institute, a Cuban epidemiological research institute	Cuba	Conjugate vaccine	Phase I study, open, sequential, and adaptive for evaluating the safety, reactogenicity and explore the immunogenicity of the prophylactic Vaccine Candidate FINLAY-FR-2 anti-SARS-CoV-2 (COVID-19)	Phase II study, multicenter and adaptive for evaluating the immunogenicity, safety, and reactogenicity of the Anti-SARS Prophylactic Vaccine Candidate—CoV-2, FINLAY- FR-2 (COVID-19)	Phase III clinical trial, multicenter, adaptive, parallel-group, randomized, placebo-controlled, double-blind study to evaluate the efficacy, safety, and immunogenicity of vaccination against SARS-CoV-2 with 2 doses of FINLAY-FR-2 and a heterologous scheme with 2 doses of FINLAY-FR-2 and a booster dose with FINLAY-FR-1A (COVID-19)	The final results of the Phase III trials in Cuba show efficacy against the symptomatic disease of 71.0% against the beta and delta strains, while a third dose of Soberana Plus increased the efficacy up to 92.4%.	3 trials in 1 country: Cuba
AZD1222 The University of Oxford, [78]	OxfordAstraZeneca	UK	Modified chimp adenovirus vector (ChAdOx1)	phase I/II (1090), Interventional, single-blinded, randomised, multi-centre study to determine efficacy, safety, and immunogenicity	A phase II/III (12,390), Interventional, randomised, single-blinded study, study to determine the efficacy, safety, and immunogenicity	Phase III (30,000) Interventional; randomized, placebo-controlled study for efficacy, safety, and immunogenicity. Brazil (5000)	Phase I-II (543) Neutralizing antibodies were detected after a booster dose was given on day 56. Side effects included pain at the injection site, headache, fever, chills, and muscle aches, acetaminophen was allowed for some participants to increase tolerability.	20 in the UK, São Paulo
BNT162b2 [79]	BioNTech Fosun Pharma Pfizer	Germany, United States of America	mRNA	Phase ½ (45), placebo-controlled, observer-blinded dose-escalation study, study on safety, tolerability, and immunogenicity.	Phase II/III (43,998), Interventional, placebo-controlled, Randomised, Observer-blind, dose-finding study, study on safety, tolerability, immunogenicity, and efficacy.	Phase III (44,820) Randomized, placebo-controlled	Phase III A two-dose regimen of BNT162b2 (30 μg per dose, given 21 days apart) was found to be safe and 95% effective against COVID-19. The vaccine met both primary efficacy endpoints, with more than a 99.99% probability of a true vaccine efficacy greater than 30%.	152 in the USA, Argentina, Brazil, South Africa, Turkey, and Germany
QazCovid-in is commercially known as QazVac [80]	Research Institute for Biological Safety Problems	Kazakhstan.	inactivated virus vaccine	Phase I/II (244), interventional, Randomized, Blind, Placebo-controlled Phase- I Study and Randomized, Open Phase Phase-ii Study of QAZCOVID-IN^®^- COVID-19 Inactivated Vaccine	Phase I/II (244), interventional, Randomized, Blind, Placebo-controlled Phase- I Study and Randomized, Open Phase Phase-ii Study of QAZCOVID-IN^®^- COVID-19 Inactivated Vaccine	Phase III (3000), Interventional, Multicenter, Randomized, Blind, Placebo-controlled Clinical Study. Study on safety, efficacy, and immunogenicity.	the trial is almost 50% completed and “people who have received [the] vaccine feel well; there have been no side-effects and the effectiveness of the vaccine is high. “QazCovid-in^®^ vaccine was safe and well-tolerated and induced predominantly mild adverse events; no serious or severe adverse events were recorded in both trials.”	2 trials in Kazakhstan (88)
Minhai COVID-19 vaccine trademarked as KCONVAC [81,82]	Minhai Biotechnology Co., and Shenzhen Kangtai Biological Products Co., Ltd.	China	inactivated virus vaccine	Phase I (180), interventional, Randomized, Double-blind, Placebo Parallel-controlled, study for safety and immunogenicity	Phase II (1000), interventional, randomised, Double-blind, Placebo Parallel-controlled, study for safety and immunogenicity.	Phase III (28,000), Interventional, Randomized, Double-blind, Placebo-controlled, study for efficacy, safety, and immunogenicity.	KCONVAC induced a significant antibody response. 87.5% (21/24) to 100% (24/24) of participants in the phase I trial and 83·0% (83/100) to 100% (99/99) of participants in the phase II trial seroconverted for neutralising antibody to live virus, neutralising antibody to pseudovirus, and RBD-IgG after receiving two doses.	5 trials in China (88)
COVIran Barekat [81,82]	Shifa Pharmed Industrial Co.,	Iran	inactivated virus vaccine	Phase I (56), randomized, double-blind, parallel arms, placebo-control clinical trial, study for safety and immunogenicity	Phase II/III (20,000), randomized, double-blind, parallel arms, placebo-controlled clinical trial, study for efficacy and immunogenicity	Phases II and III of the clinical trials were combined	Phase I–II only mild adverse effects were registered except for one case of hypotension, one case of level-2 headache, and one case of diminution of platelets that didn’t need medical care. Conventional Virus Neutralizing Test (cVNT) is reported to have shown 93.5% immunogenicity (95% confidence interval: 88.4–99.6%). Phase II “the serum of the people who received the vaccine has 93.5% power to neutralize the virus; thus, meaning the vaccine has a very good efficacy that will be shown after the end of the third phase”.	4 trials in Iran (88)
Chinese Academy of Medical Sciences COVID-19 vaccine [81,82]	Chinese Academy of Medical Sciences.	China	inactivated virus vaccine	Phase I/II (942), Interventional, randomized, double-blinded, and placebo-controlled, study for safety and immunogenicity	Phase I/II (942), Interventional, randomized, double-blinded, and placebo-controlled, study for safety and immunogenicity	Phase III (34,020), Interventional, randomized, double-blinded, placebo-controlled, study for efficacy, safety, and immunogenicity	Phase III (Brazil) In the age group 18–59 years old, the efficacy of the vaccine was 50.66%, and 50.11% in the age group 60 years and above. The overall incidence of adverse reactions was 77.10% in the vaccine group and 66.37% in the placebo group, with local and collective adverse reactions as the main; πThe incidence of local adverse reactions was 62.14% in the vaccine group and 35.32% in the placebo group. The incidence of systemic adverse reactions was 58.45% in the vaccine group and 56.91% in the placebo group; The most common symptoms were pain at the inoculation site, headache, fatigue, and myalgia;	Phase III 3 trials in UAE, Brazil, and Malaysia. Phase II 2 trials in China. Phase I 2 trials in China. (88)
ZyCoV-D [83]	Indian-based pharmaceutical company Cadila Healthcare with support from the Biotechnology Industry Research Assistance Council.	India	DNA plasmid	Phase I (48), single-center, open-label, non-randomized, study for safety and immunogenicity.	Phase II (1000), as part of the adaptive Phase I/II multi-centric, dose-escalation, randomised, double-blind placebo-controlled method.	Phase III (28,216), Interventional, randomized, multi-centre, double-blind, placebo-controlled, study to evaluate the efficacy, safety, and immunogenicity.	Phase III Interim results from Phase III showed a primary efficacy of 66.6 % for symptomatic RT-PCR positive cases. This vaccine had already exhibited robust immunogenicity and tolerability and safety profile in the adaptive Phase I/II clinical trials carried out earlier.	3 trials in India (88)
FAKHRAVAC (or MIVAC) [84]	Organization of Defensive Innovation and Research	Iran	inactivated virus vaccine	Phase I (135), Randomized, double-blind, placebo-controlled trial with factorial design, study on safety and immunogenicity.	Phase II (500), Randomized, double-blind, controlled trial with parallel design, study on safety and immunogenicity.	Phase III (41,128), Randomized, non-inferiority, double-blind, controlled trial with parallel design. Study on safety and efficacy.	It is not yet known how safe and effective FAKHRAVA is. The defense ministry, which developed it, was having trouble finding enough participants for those trials, due to the lack of demand for vaccines which are domestic manufacturers.	3 trials in Iran. (88)
COVAX-19 also known as SpikoGen [81,83]	Australian-based company Vaxine and Iran-based company CinnaGen	Australia/ Iran	protein subunit vaccine	Phase I (40), Interventional, A Randomised, Controlled, parallel, non-blind study. Study on safety and Immunogenicity	A Phase II (400), Randomized, parallel, Two-armed, Double-blind, Placebo-controlled Trial to Evaluate the Safety, Tolerability, and Immunogenicity	Phase III (16,876), Interventional, Randomized, Two-armed, Double-blind, Placebo-controlled Trial to Evaluate the Efficacy and Safety	Vaccine passes 60% efficacy bar. SpikoGen has a favourable safety profile.	5 trials in Iran, and Australia. (88)
Razi Cov Pars [81,83]	Razi Vaccine and Serum Research Institute.	Iran	protein subunit vaccine	Phase I (133), a randomized, double-blind, placebo-controlled trial, study on safety and immunogenicity.	Phase II (500), two parallel groups, randomized, double-blind, placebo-controlled trial, study on safety and immunogenicity	Phase III (41,128), two parallel and equal groups, randomized, double-blind, non-inferiority design; study on safety and efficacy.	People who received COV Pars did not have any side effects. COV Pars vaccine can not only induce antibodies but can also activate cellular immunity. 14,000 people received the shot that was completely resistant to the Wuhan variant, but with the advent of the delta strain, the effectiveness of the vaccine was slightly reduced and 20 percent of those who received the vaccine had mild symptoms of the disease, which disappeared within one to two days.	3 trials in Iran (88)
Turkovac [85]	Health Institutes of Turkey and Erciyes University	Turkey	inactivated vaccine	Phase I (44), Interventional, double-blind, double dose, parallel, randomized vaccination study. Study on safety and immunogenicity.	Phase II (250), Interventional, Randomised, Parallel, Study on Efficacy, Immunogenicity, and Safety	Phase III (40,800), Interventional, randomized, double-blinded, Parallel, multi-center, active-controlled phase III clinical trial Study on Efficacy, Immunogenicity, and Safety	It is not yet known how safe and effective Turkovac is.	6 trials in Turkey (88)
Sinopharm CNBG [86]	China National Biotec Group (CNBG)	China	recombinant protein subunit vaccine	Phase I/II (690), Interventional, non-randomized, parallel, single-blinded study. Study on Safety, Reactogenicity, and Immunogenicity.	phase II (800), Interventional, non-randomized, open-label, clinical trial to evaluate the safety and immunogenicity	Phase III (12,000), Interventional, randomized, parallel, quadruple-blinded study. Study on safety, efficacy, and immunogenicity.	Phase III: A large multi-country Phase III trial has shown that 2 doses, administered at an interval of 21 days, have an efficacy of 79% against symptomatic SARS-CoV-2 infection 14 or more days after the second dose. Vaccine efficacy against hospitalization was 79%.	18 trials in Iran, China, Pakistan, Argentina, China, Mozambique, Thailand, UAE, Peru, Bahrain, Egypt, and Jordan, (88)
Corbevax [87]	Texas Children’s Hospital at the Baylor College of Medicine	Houston, Texas	protein subunit vaccine	Phase I/II (360), Prospective, multicentre study. Study on safety and immunogenicity.	Phase I/II (360), Prospective, multicentre study. Study on safety and immunogenicity.	Phase III (1268), study on immunogenicity and safety.	Phase III Corbevax was found to be safe, well-tolerated, and immunogenic. CorbeVax demonstrated a superior immune response than the COVISHIELD vaccine when assessed for Neutralizing Antibody (nAb) Geometric Mean Titers (GMT) against the Ancestral-Wuhan strain and the globally dominant Delta variant. CorbeVax vaccination also generated a significant Th1 skewed cellular immune response. Phase III clinical trial results indicate vaccine effectiveness of >90% for the prevention of symptomatic infections. CorbeVax nAb GMT against the Delta strain indicates vaccine effectiveness of >80 percent for preventing symptomatic infections based on published studies. While none of the subjects who took CorbeVax had serious adverse events, CorbeVax had 50 percent fewer adverse events than COVISHIELD.	2 trials in India (88)

**Table 2:** Vaccine candidates that went to phase I–II but were paused.

Vaccine Candidate	Developer	Country	Technology Used	The Phase in Which It Is Paused
GX-19N [88,89]	Genexine	South Korea	DNA-based vaccine	Phase I/II
COVAC1 [88,90]	Imperial College London partnering with Morningside Ventures	UK	“self-amplifying” RNA vaccine	Phase I/II
MRT5500 [88,91]	Sanofi collaborated with Massachusetts-based Translate Bio	France	mRNA vaccine	Phase I/II
CORVax12 [88,92]	OncoSec Immunotherapies	New Jersey	a loop of DNA	Phase I
V591 [88,93]	Merck acquired the Austrian firm Themis Bioscience in June 2020 to develop their vaccine, which had been originally developed at Institut Pasteur.	America	a weakened measles virus that carries a gene for the coronavirus spike protein.	Phase I
V590 [88,93]	Merck partnered with IAVI	America	viral vector vaccine, based on vesicular stomatitis viruses	Phase I
AdCOVID [88,94]	Maryland-based Altimmune	United States of America	Intranasal adenovirus type 5-vectored vaccine encoding the receptor-binding domain (RBD) of the SARS-CoV-2 spike protein.	Phase I
IVX-411 [88,95]	ICOSAVAX	Seattle, USA	Protein subunit	Phase I/II
CoVepiT [88,96]	OSE Immunotherapeutics	Belgium	Optimized peptides selected to induce a lasting sentinel T lymphocyte immune response against SARS-CoV-2 in barrier tissues, the respiratory tract, and the lung.	Phase I
QazCoVac-P [88]	Research Institute for Biological Safety Problems	Kazakhstan	Protein subunit	PhaseI/II
NBP2001 [88]	SK Bioscience	South Korea	Protein subunit	Phase I
V451 [88,97]	The University of Queensland and CSL	Australia	Protein subunit Molecular Clamp vaccine technology	Phase I
Fakhravac [88,98]	Organization of Defensive Innovation and Research	Iran	inactivated coronaviruses	Phase II

## Data Availability

This study did not report any data.
